# Endoplasmic reticulum stress mediates the myeloid-derived immune suppression associated with cancer and infectious disease

**DOI:** 10.1186/s12967-022-03835-4

**Published:** 2023-01-02

**Authors:** Xiaoli Lou, Deyong Gao, Liyuan Yang, Yue Wang, Yanqiang Hou

**Affiliations:** 1 Department of Clinical Laboratory, Songjiang Hospital Affiliated to Shanghai Jiao Tong University School of Medicine, Shanghai, 201600 China; 2 Department of Infectious Disease, Songjiang Hospital Affiliated to Shanghai Jiao Tong University School of Medicine, Shanghai, 201600 China

**Keywords:** Myeloid-derived suppressor cells, Endoplasmic reticulum stress, Immune suppression, Cancer, Leprosy and tuberculosis

## Abstract

Myeloid-derived suppressor cells (MDSCs), which are immature heterogeneous bone marrow cells, have been described as potent immune regulators in human and murine cancer models. The distribution of MDSCs varies across organs and is divided into three subpopulations: granulocytic MDSCs or polymorphonuclear MDSCs (G-MDSCs or PMN-MDSCs), monocytic MDSCs (M-MDSCs), as well as a recently identified early precursor MDSC (eMDSCs) in humans. Activated MDSCs induce the inactivation of NK cells, CD4+, and CD8+ T cells through a variety of mechanisms, thus promoting the formation of tumor immunosuppressive microenvironment. ER stress plays an important protecting role in the survival of MDSC, which aggravates the immunosuppression in tumors. In addition, ferroptosis can promote an anti-tumor immune response by reversing the immunosuppressive microenvironment. This review summarizes immune suppression by MDSCs with a focus on the role of endoplasmic reticulum stress-mediated immune suppression in cancer and infectious disease, in particular leprosy and tuberculosis.

## Introduction

Myeloid-derived suppressor cells (MDSCs) are immature myeloid suppressor cell populations that are derived from the bone marrow. MDSCs accumulate and exert immune suppressive effects during pathologic conditions such as cancer, inflammation, infection, autoimmune disease, and obesity [1]. The MDSCs suppress T cell activation by downregulation of L-selectin and sequestration of cysteine, which the T cells cannot synthesize spontaneously and that they require to become activated. The development, expansion, and activation of MDSCs were triggered by the tumor microenvironment, particularly the immune microenvironment, and regulated by differential intracellular signaling molecules [2]. The microenvironment during these pathologic conditions is characterized by a low pH, hypoxia, nutrient deprivation, and free radicals. This microenvironment disrupts protein folding, which triggers cellular “endoplasmic reticulum (ER) stress” [3]. ER stress impacts inflammatory and tumor microenvironment-induced immune suppression [4–6]. Furthermore, tumor cells can transmit ER stress to immune cells recruited to inflammatory tissues [7, 8]. Most noteworthy, there is compelling evidence that ER stress can transform immune cell populations into immunosuppressive phenotypes [6, 9], with MDSCs from cancer patients and tumor-bearing mice producing a robust ER stress response. Many factors can induce ER stress in MDSCs. Reactive oxygen species (ROS), one of the main inducers of the ER stress response, are a significant product of MDSCs [10]. Lipids can also induce ER stress [11], with lipid accumulation associated with MDSCs [12].

Currently, MDSCs are becoming the main immuno-therapeutic targets. How ER stress regulates the biological properties of MDSCs in the tumor microenvironment is critical for MDSCs-targeted immunotherapy. This review summarises the ER stress effect on the immunosuppressive function of MDSCs in different kinds of tumors and infectious diseases, focusing on *Mycobacterium leprae* and *Mycobacterium tuberculosis* infections. We also summarized investigated molecules as the immunotherapy targets aiming to provide a more comprehensive theoretical basis for targeted MDSCs immunotherapy in the clinic.

## MDSCs, their expansion, roles, and mechanisms in immunosuppressive function in pathological conditions

The terminology of MDSCs was first defined in 2007 and referred to the origin and the suppressive function of these cells. On physiological conditions, hematopoietic stem cells (HSCs) first develop into common myeloid progenitors (CMPs) and then into immature myeloid cells (IMCs). IMCs further differentiate into mature functional granulocytes, macrophages, and dendritic cells (DCs). However, during pathological conditions, IMCs differentiate into MDSCs within the bone marrow and then migrate to peripheral tissues [13] .

MDSCs are a heterogeneous population composed of monocytes, polymorphonuclear leukocytes and immature myeloid cells. MDSCs are broadly divided into three subgroups: granulocytic MDSCs or polymorphonuclear MDSCs (G-MDSCs or PMN-MDSCs), monocytic MDSCs (M-MDSCs) [14], as well as a recently identified early precursor MDSC (eMDSCs) in humans [15]. In mice, Gr-1 and CD11b are used to identify MDSCs. Ly6G and Ly6C are used to distinguish M-MDSCs (CD11b^+^Ly6G^−^Ly6C^high^) from G-MDSCs (CD11b^+^Ly6G^+^Ly6C^low^) [16]. In humans, the common MDSC phenotype is CD11b^+^HLA-DR^−/low^. CD33 is the common myeloid marker for humans, while CD14 and CD15 are used to distinguish M-MDSCs (CD11b+HLA-DR^−/low^CD33+CD15−CD14+), G-MDSCs (CD11b^+^HLA-DR^−/low^CD33^+^CD15^+^CD14^−^), and eMDSCs (CD11b^+^HLA-DR^−/low^CD33^+^CD15^−^CD14^−^). It is difficult to identify G-MDSCs from neutrophils in mice or humans, as they have a similar phenotype. However, the two cell populations can be distinguished by density gradient centrifugation, which has limitations [15, 17]. Recently, it is found that Lectin-type oxidized LDL receptor-1 (LOX-1) is a unique surface marker of human G-MDSCs, which can be used as a distinguish marker of G-MDSCs. Meanwhile, S100A9 has been used to refine identification of M-MDSCs in human [18]. Work from several groups has demonstrated that the key immunosuppressive feature does not distinguish MDSCs from conventional myeloid cells during inflammation [19]. A combination of molecular markers is considered being the most accurate means by which to identify different subtypes of MDSCs, with the caveat that different methods for collection and analysis of MDSCs can influence outcomes.

MDSCs accumulate during pathological conditions such as infection, inflammation, traumatic stress, and cancer [20]. The expansion of MDSCs to the pathological sites is induced by factors primarily produced by tumors or bone marrow stromal cells. Macrophage CSF (M-CSF), granulocyte/macrophage colony-stimulating factor (GM-CSF), granulocyte CSF (G-CSF), IL-6, IL-1, β-fibroblast growth factor (β-FGF) and vascular endothelial growth factor (VEGF) affect the mobilization and the expansion of MDSCs [21–23]. The primary transcription factor that regulates expansion and activation of MDSCs is STAT3, which is a downstream target of phosphorylated Janus kinases (JAKs). Essential genes for MDSC survival and proliferation are Bcl-XL, MYC, survivin, and cyclin D1, which are upregulated by STAT3. S100A8/S100A9 expression is activated by STAT3, which then modulates interferon regulatory factor-8 (IRF-8), a negative regulator of MDSCs. Further, miR-155 and miR-21 induce MDSCs by reduction of the negative regulators (SHIP-1 and PTEN), increasing STAT3 activation [24–26].

When MDSCs expanded to the tumor or inflammatory sites, activation signals were launched and endowed MDSCs to carry out the inhibitory function. The NF-κB signaling pathway is essential to MDSC activation. IL-1β activates MDSC recruitment and promotes IL-6 and TNF-α production through the NF-κB pathway. M-MDSCs from cancer patients produced a high level of TGF-β secretion when treated with PGE_2_ activating p38 MAPK/ERK signaling [27, 28]. For G-MDSCs, eIF2 and eIF4 were related to ER stress, mTOR and MAPK pathway upregulation [29]. T cell immunosuppression is due to the depletion or sequestration of amino acids. The stress of low extracellular amino acid levels promotes the activation of metabolic sensor (GCN2 kinase, FATP2, and AMPK) and the accumulation of metabolic waste products within the tumor microenvironment (TME) [2]. Furthermore, MDSC-mediated immunosuppression can be induced by the metabolic conversion of the amino acid l-arginine by arginase 1 (ARG1) or by the production of inducible nitric oxide synthase (iNOS). The iNOS degrades l-arginine to produce nitric oxide (NO) and citrulline. ARG1 uses l-arginine as a substrate to produce l-ornithine and urea. As a result of l-arginine starvation, T-cell proliferation and the synthesis of T-cell effector molecules are impaired, leading to severe T-cell dysfunction [21, 22]. Further, NO production by iNOS prevents IL-2 production by activated leukocytes in that the stability of IL-2-encoding mRNA is impaired. The loss of l-cystine and l-cysteine inhibits activated T cell synthesis of the anti-oxidant glutathione, which impairs proliferation and activation of T cells [23]. In addition, MDSCs induce tryptophan depletion via indoleamine-pyrrole 2,3-dioxygenase (IDO). IDO catalyzes extracellular l-tryptophan to kynurenine. Tryptophan depletion and kynurenine exposure hinders T cell proliferation and facilitates the expansion of regulatory T cells [2]. MDSCs also produce reactive nitrogen species (RNS) and ROS as well as other suppressive molecules that blunt TCR signaling and reduce T cell survival [30]. Furthermore, persistent ER stress promotes tumor progression by affecting malignant cells and infiltrated MDSCs. The regulation of MDSC expansion and suppressive function was shown in Table 1.

**Table 1:** Regulation of MDSC expansion and suppressive mechanisms

Function	Signaling pathways	References
Mobilization and expansion	Granulocyte/macrophage colony-stimulating factor (GM-CSF), macrophage CSF (M-CSF), granulocyte CSF (G-CSF), IL-6, IL-1β, beta-fibroblast growth factor (β-FGF), and vascular endothelial growth factor (VEGF)	[21–23]
	JAKs/STATs/Bcl-XL,MYC, survivin, cyclin D1 signaling	[19]
	STAT3/S100A8, S100A9/IRF-8 signaling	[24, 25]
Activation	miR-155, miR-21 down-modulate SHIP-1/PTEN and increase STAT3 activation	[26]
	NF-κB/IL-1β, IL-6 and TNF-α pathway	[27, 28]
	PGE_2_/p38 MAPK/ERK/TGF-β	[64, 65]
Immunosuppression	GCN2 kinase, FATP2 and AMPK	[27–30]
	ARG1 utilizes l-arginine as a substrate to produce l-ornithine and urea	[21]
	iNOs degrades l-arginine to produce nitric oxide and citrulline	[22]
	iNOS prevents IL-2 production in activated leukocytes by impairing the stability of interleukin 2 (IL-2)-encoding mRNA	[24]
	MDSCs sequester l-cystine by expressing cystine/glutamate antiporter xCT (XCT; *SLC7A11*)	[25]
	MDSCs induce depletion of tryptophan via indoleamine-pyrrole 2, 3-dioxygenase (IDO) which catalyzes reduction of extracellular l-tryptophan to produce kynurenines	[2]
	MDSCs produce RNS or ROS, chemokines, cytokines and other suppressive mediators to blunt TCR signaling and inhibit T cell survival persistent ER stress prolonged immunosuppression effects of MDSCs	[2, 30]

In addition, ferroptosis can promote anti-tumor immune response by reversing immunosuppressive microenvironment. A comprehensive index of ferroptosis and immune status (CIFI) was concluded from twenty-seven prognostic ferroptosis- and immune-related signatures in hepatocellualr carcinoma, which could predict a subgroup of patients with a worse prognosis. These patients have higher fractions of cancer-associated fibroblasts (CAFs) and MDSCs [31]. A manganese porphyrin-based metal-organic framework (Mn-MOF), FAP gene-engineered tumor cell-derived exosome-like nanovesicles (eNVs-FAP), NC06 or the Dihydroartemisinin (DHA) were explored to treatment hypoxic tumors or as the a candidate tumor vaccine, which was designed to reduce the number of MDSCs by targeting ferroptosis [32–37]. It is indicated that ferroptosis inducer by controlling MDSC polarization or population is a promising immuno-therapeutic strategy [38]. The ferroptosis-based immunotherapy targets affecting the MDSCs population in different kinds of cancers was shown in Table 2.

**Table 2:** The prognostic indicator and ferroptosis-based immunotherapy targets affecting the MDSCs population in different kinds of cancers

Diagnostic markers or therapeutic targets	Cancer type	Signalling pathway	Immune cell populations	References
Comprehensive index of ferroptosis and immune status (CIFI)	Hepatocellular carcinoma (HCC)	–	A subgroup of patients with high CIFI value have higher fractions of cancer-associated fibroblasts (CAFs) and MDSCs	[31]
A manganese porphyrin-based metal-organic framework (Mn-MOF) for enhanced SDT and ferroptosis	Hypoxic tumors	Self-supply oxygen (O_2_) and decrease GSH	Increase the numbers of activated CD8+ T cells and matured dendritic cells and decrease the numbers of MDSCs in tumor tissues	[32]
FAP gene-engineered tumor cell-derived exosome-like nanovesicles (eNVs-FAP) as a tumor vaccine	A candidate tumor vaccine targeting both the tumor parenchyma and the stroma	Promote tumor ferroptosis by releasing interferon-gamma (IFN-γ) from CTLs	Induce strong and specific cytotoxic T lymphocyte (CTL) immune responses and deplete FAP + CAFs (cancer-associated fibroblasts)	[33]
HMGB1, ferroptosis inducer	Head and neck squamous cell carcinoma (HNSCC)	GPX4/RSL3/HMGB1	The occurrence of ferroptosis reduce the number of MDSCs and TAMs (M2) and increase CD4+ and CD8+ T cells in tumor microenvironment.	[34]
NC06 targeting ASAH2 (*N*-acylsphingosine amidohydrolase)	Colon carcinoma	NC06/ASAH2/p53/Hmox1/ROS	Inhibition of ferroptosis decreased NC06-induced MDSC death	[35]
Dihydroartemisinin (DHA)	PDAC	P53/ALOX12	Decrease the suppressive expansion of M2 and MDSCs and increase the population of CD8+ T cells, NK cells and NKT cells in the tumor tissues of the tumor-bearing mice	[36]
Pam3CSK4 targeting Runx1	Hepatocellular carcinoma	TLR2/Runx1/ROS	Reduce the expression of CD11c, F4/80, CD80/CD86 and MHC-II in MDSC and recover T cell function	[37]
MGST1	Uterine corpus endometrial carcinoma (UCEC)	MGST1 interacts with several ferroptosis-related proteins	Overexpression of MGST1 was accompanied by lower levels of NK cell and CD8+ T cell infiltration, higher MDSCs infiltration and different immunocytes with corresponding markers	[38]

## Unfolded protein response (UPR) and ER stress

ER is a closed plumbing system within the eukaryotic cytoplasm. It is divided into rough ER and smooth ER. The rough ER performs functions related to membrane synthesis and secretion of proteins and is widespread in cells with high secretory capacity. The smooth ER is responsible for the synthesis and transport of lipids. ER is a crucial cell organelle that is involved in the regulation of calcium homeostasis, protein synthesis, lipid metabolism, post-translational modification, transport, and is an essential organelle for synthesis and folding of secreted and transmembrane proteins. However, cellular stressors such as hypoxia, nutrient deficiency, and Ca^2+^ homeostatic induced ER function disorder which lead to unfolded or misfolded protein accumulation in the ER lumen. If proteins are not properly folded, they are ubiquitinated on the ER membrane and subsequently degraded in a process known as ER associated protein degradation (ERAD). When accumulated misfolded proteins are not eliminated by ERAD, the ER activates the unfolded protein response (UPR). UPR signaling has important roles in immunity, inflammation, and different types of cancer [39, 40].

The UPR has three important sensors: inositol requiring enzyme 1 (IRE1ɑ), protein kinase RNA-activated (PKR)-like ER kinase (PERK), and activating transcription factor 6 (ATF6), which are transmembrane proteins associated with the ER [41]. As well, glucose-regulated protein 78 (GRP78), also referred to Bip or HSPA5, is a key ER chaperone that binds accumulated unfolded/misfolded proteins within the ER lumen. This binding process promotes protein folding and trafficking. Thus, GRP78 is a marker of ER stress and plays an important role in combating ER stress of solid tumor cells [42, 43].

## ER stress or exposure to tumor-related ER stress augments the immunosuppressive potential of MDSCs

**Fig. 1:**
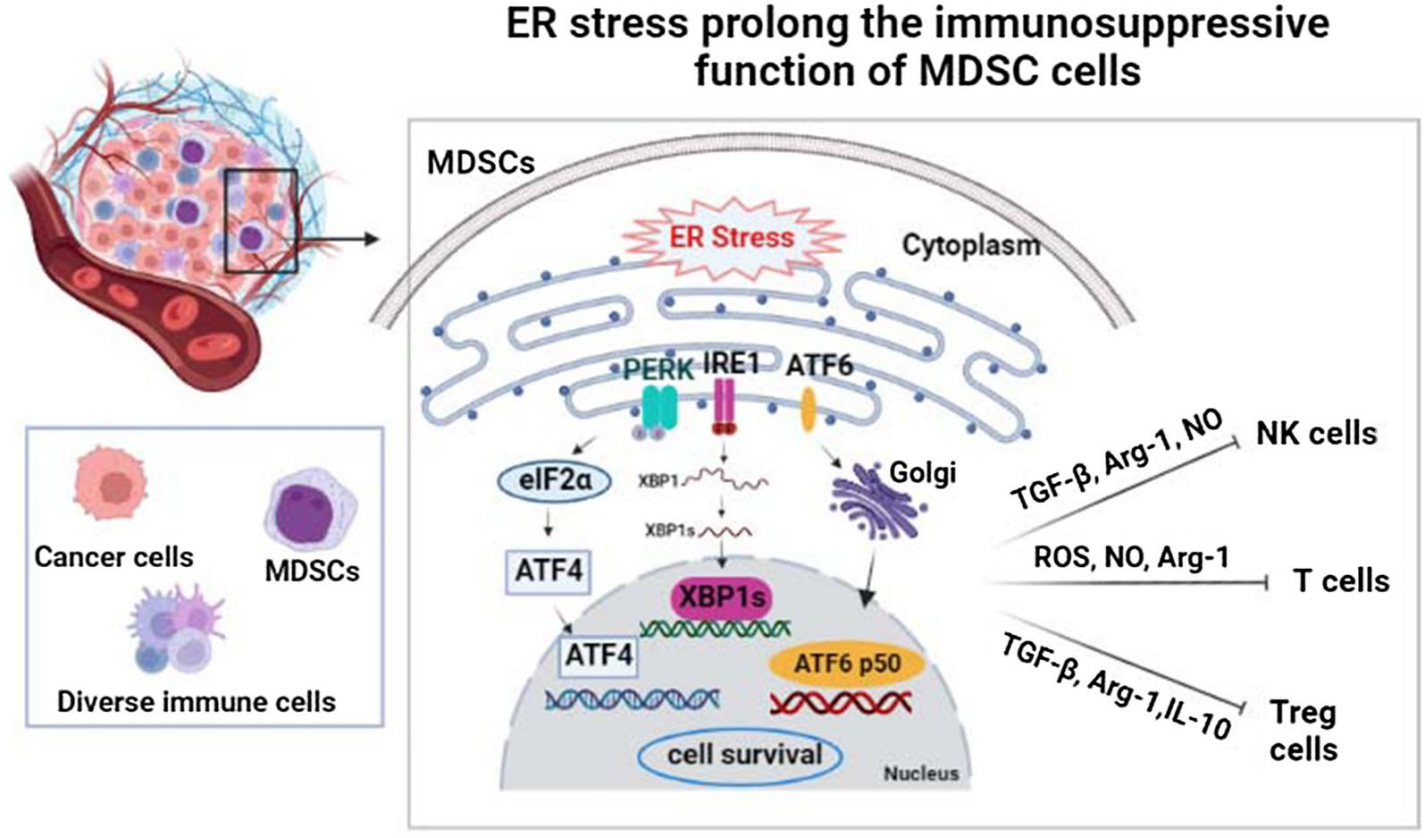
ER stress prolong the immunosuppressive function of MDSC cells. MDSCs induce the inactivation of NK cells, CD4+, CD8+ T, and Treg cells through the secretion of TGF-β, NO, and Arg-1. In addition, hypoxia, nutrient starvation, low pH, and increased levels of free radicals in the tumor microenvironment trigger the activation of ER stress sensors, such as IRE1ɑ, PERK, and ATF6, which results in the activation of ER stress of MDSCs. ER stress prolongs the survival of MDSCs and thus aggravates the immunosuppression in tumors

The tumor microenvironment (TME) comprises tumor cells, immune cells, the extracellular matrix and chemokines, cytokines, growth factors, and extracellular vesicles. MDSCs, DCs, and macrophages accumulate within infectious or tumor microenvironments in which hypoxia, nutrient starvation, low pH, and increased levels of free radicals trigger a state of ER stress in cancer cells and in infiltrating myeloid cells. The UPR response triggered by ER stress protects cells from damage. However, when damage is excessive, UPR signals self-destruction, which removes bacteria and prevents further damage. In response to ER stress, cancer cells and MDSCs activate the UPR to promote cell survival and adaptation during adverse environmental conditions [2]. MDSC infiltrates tumor tissues and displays immunosuppression function by suppressing NK cells, T cells, and Treg cells. MDSCs could also display ER stress to survive in the hypoxia-induced tumor microenvironments. The survival MDSCs could produce Arg1, NO, and TGF-β and play roles in immunosuppression [21, 22]. Thus ER stress plays an essential protecting role in the survival of MDSC, which aggravates the immunosuppression in tumors (Fig. 1). The role of ER stress in immune modulation has not fully characterized, but the effects of ER stress on MDSCs during infection and cancer are described below.

## Immunosuppressive effects of ER stress and UPR on MDSCs in infectious disease

When inflammation or infection occurs, MDSCs rapidly expand and travel to the injury sites and regulate the host’s immune system. Therefore, it is crucial to have a thorough understanding of immunomodulatory mechanisms of infection and inflammatory diseases, which may also assist in exploring therapeutic targets. The current data highlight the contribution of ER stress to MDSC immuno-suppression function. Indeed, ER stress can also occur in infectious diseases. Therefore, exploring the role of ER stress in MDSC regulating inflammation helps overcome bacterial infection. By now, the research concerning the interaction of ER stress and MDSCs in infectious disease mainly focuses on Mycobacterium leprae and Mycobacterium tuberculosis infections.

### ER stress activates MDSCs and mediates immunosuppression during *Mycobacterium leprae* and *Mycobacterium tuberculosis* infections

Leprosy and tuberculosis are caused by intracellular *M. leprae* and *M. tuberculosis*, respectively. Various immune system components such as M1 and M2 macrophages, natural killer (NK) cells, DCs, and diverse subtypes of lymphocytes are involved in these infections. Infection can also trigger the accumulation of MDSCs at inflammatory sites [44–46]. However, *M. tuberculosis* and *M. leprae* can escape and evade the host’s innate immune system [47, 48]. Tuberculoid leprosy (T-LEP) is self-limiting with few bacilli. The host response is a Th1 type. Lepromatous leprosy (L-lep) is a progressive form of disease that is characterized by a high bacillary load within macrophages. The host response to L-lep is a Th2 type [49], with the number of MDSC greater in L-lep patients than in T-lep patients. MDSCs from L-lep patients suppress T-cell proliferation of *M. leprae*-specific T cells and reduce the production of IFN-γ, which allows bacterial growth and disease progression. Therefore, immunosuppression by MDSCs may worsen *M. leprae* infection and contribute to the progression of leprosy [44, 45, 50].

GM-CSF and M-CSF drive the expansion of myeloid immune cells within the bone marrow and spleen. MDSCs can be recruited and activated by many factors, such as the proinflammatory cytokines IL-1β, IL-6, and IFN-γ. ER stress can also activate MDSCs and trigger these cells to produce iNOS, ROS, and Arg-1, that are immune suppressive [51–53]. Kelly-Scumpia et al. [54] found an increase in immature myeloid cells displaying a granulocytic MDSC cell-surface phenotype (HLA-DR-CD33+CD15+) and T-cell suppressive activity in the blood of patients with disseminated/progressive leprosy when compared to self-limited T-Lep. In terms of mechanism, ER stress significantly regulates the T cell inhibitory activity of MDSCs. Further, ER stress promotes IL-10 secretion, which contributes to MDSC activity and highlights the role of ER stress and IL-10 in MDSC-mediated effects during human *M. leprae* infection [55]. Further, MDSC ER stress can be caused by circulating IL-1α, IL-6, and IFN-γ [53] in L-lep and tuberculosis infections. These cytokines also cause ER stress in macrophages, DCs, and T cells in L-lep patients, suggesting ER stress may be another factor contributing to the exacerbation of leprosy and tuberculosis [6, 54]. Uncontrolled bacterial growth worsens the ER stress in MDSCs, resulting in increased production of IL-10 and enhanced immunosuppressive activity [5, 56]. Taken together, MDSC mediated immune-suppression is a leading cause of *M. leprae* and tuberculosis infection, with ER stress activating MDSC immunosuppressive activity. Crispr-cas9, ZFNs, and TALENS are new genetic tools [55, 57] that can block IRE1α and XBP1 signaling and stabilize the ER of MDSCs. Previous studies have shown that reducing the expression of CHOP in MDSCS can promote immune activity and stimulate T cells [8]. Therefore, targeting the UPR could regain or reduce ER stress in tuberculosis and leprosy, thereby reducing the immunosuppressive activity of MDSCs [58, 59]. Breaking ER homeostasis in MDSC may be a potential strategy to combat and eradicate leprosy and tuberculosis.

## Immunosuppressive effects of ER stress and UPR on MDSCs in cancers

MDSC was initially described as immunosuppressive myeloid cells that evade cancer. The MDSCs, which accumulate in tumor-bearing mice and cancer patients, are site-specific inflammatory and immunosuppressive agents that contribute to cancer progression in different cancers. MDSCs accumulated in the TME under chronic inflammation conditions and cancer contributed to the growth of tumors. Furthermore, the population of immunosuppressive MDSCs decreased after radiotherapy. Thus, preventing MDSC development and/or interfering with their immunosuppressive functions in cancer could reduce immunosuppression, thereby increasing antitumor immunity. In this part, we will discuss ER stress-activated MDSCs and enhanced immunosuppression, which may serve as targets in immunotherapy for different kinds of tumors.

### ER stress and MDSCs as therapeutic targets for ulcerative colitis and colorectal cancer

Colorectal cancer (CRC) is one of the primary causes of cancer-related deaths globally, with more than 2.2 million new cases projected by 2030 [60]. Ulcerative colitis is a chronic colon inflammation, a complex, recurrent, and remitting form of intestinal inflammation [61]. For ulcerative colitis and CRC, MDSCs are a main component of the inflammatory microenvironment with infiltration of the intraepithelial and lamina propria layers. When activated, MDSCs reduce T cell immune function and recruit tumor-associated macrophages (TAM) that down-regulate immune activity in the colonic epithelial barrier [62]. Furthermore, MDSCs secrete M-CSF and GM-CSF that recruit tumor-associated neutrophils (TANs) and TAMs in inflamed colon intraepithelial, lamina propria, and cancerous tissues [63]. In addition, studies have shown that UPR activation and ER stress are involved in colitis and tumorigenesis. During colitis, the stable status of the ER protein-folding environment is disrupted by physiologic, pathologic, or environmental injury, which results in the accumulation of misfolded proteins. When the accumulation of misfolded proteins exceeds the tolerance threshold, the ER-resident sensors trigger the UPR, resulting in transcriptionally enhanced ER protein folding capacity [64]. Colonic mucosal cells undergo apoptosis if these corrections are insufficient. However, if cells limit pro-apoptotic UPR successfully, ER stress can promote tumorigenesis [65]. Thus, continuous activation of robust ER stress sensors can confer tumorigenesis. Studies have shown that controlling robust ER stress is an effective therapeutic strategy for the prevention of colitis and tumorigenesis [5]. Feng Wang et al. demonstrated a new derivative of myricetin, (M10), to inhibit ulcerative colitis and colorectal neoplasms by weakening gross ER stress. Inhibition of ER stress-induced the UPR pathway by direct regulation of mTOR expression. Therefore, M10 may be a promising drug for chemo-prophylaxis of colitis and tumorigenesis [66]. In addition, MDSCs may be an effective therapeutic target in that emerging evidence suggests critical roles for GM-CSF and M-CSF in chronic, relapsing, and complex inflammatory states in colonic tissues [67]. MDSCs can also produce IL-6 and TNF-ɑ, which are involved in the IL-6/STAT3 pathway signaling, playing an immunosuppressive role in the tumor microenvironment [68, 69]. It has been reported that naringin inhibits MDSCs, proinflammatory mediators (GM-CSF/M-CSF, IL-6, TNF-ɑ), and the NF-κB/IL-6/STAT3 cascade in colorectal tissue, reducing the severity of colitis and colorectal adenoma. Naringin inhibits ER transmembrane proteins (GRP78, ATF6, and IRE1), as well as activated PERK, phosphorylated eIF-2α in colorectal mucosal cells. Further, naringin prevents the secretion of the ATG3, ATG5, ATG7, ATG12, ATG16, and ATG16L1 complex, thus preventing the occurrence of colitis and colorectal cancer [70].

### ER stress, MDSCs, and breast cancer

Triple-negative breast cancer (TNBC) accounts for 15.0−25.0% of all breast cancers. TNBC cells do not express the estrogen receptor (ER), the progesterone receptor (PR), or the human epidermal growth factor receptor-2 (HER-2). TNBC is an early onset and highly aggressive malignant tumor with a poor prognosis and a high distant metastasis rate [71, 72]. Activated PERK, one of the ER-membrane-resident sensors, can phosphorylate eIF2 and induce a comprehensive stress response that results in global translation inhibition and selective translation of repair proteins [73, 74]. Overexpression of P-EIF2A has been associated with tumor progression [75, 76] and a protective clinical effect [77, 78]. Thus the effect of the tumor PERK/P/EIF2A signaling pathway is controversial. In breast cancer (BC), P-EIF2A has been reported to predict disease-free survival in patients with TNBC [79]. Zou et al. reported EIF2A mRNA levels to be negatively associated with TNBC relapse-free survival and negatively related to metastasis. P-EIF2A promotes the activity of tumor-infiltrating T cells and inhibits the activity of MDSCs by inhibiting PDL1 and CXCL5, thereby regulating TNBC metastasis. The PERK/EIF2A pathway also regulates carboplatin resistance in highly metastatic TNBC.

IRE1α, one of the ER-membrane-resident sensors, remodels the TME in TNBC by increasing pericyte levels and vascular normalization while decreasing CAFs and MDSCs [80]. Matrix cellular proteins, a group of extracellular matrix (ECM) proteins, are transducers and modulators of the interaction between cells and the extracellular microenvironment. These proteins include osteopontin (OPN), thrombospondins (TSPs), osteonectin, tenascins, periostin (POSTN), and CCNs [81]. POSTN is highly expressed in many tissues but is significantly associated with the degree of tumor malignancy, metastasis, hyperplasia, and fibrosis of inflammatory tissue. POSTN is expected to become a detection index for diagnosing and treating of many tumors and inflammatory diseases. It has recently been reported that lung fibroblast-derived POSTN is an important limiting factor of metastatic breast cancer cells within the lung by promoting of the self-renewal of breast cancer stem cells [82].

Furthermore, POSTN is reported to be associated with a poor prognosis for basal-like breast cancer, with POSTN-integrin ɑvβ3 signaling required to establish a micro-environmental niche for breast cancer stem cells [83]. It is interesting to note that POSTN can also be produced by bone MDSC cells and their derived cells, which indicates that POSTN promotes MDSC-mediated pulmonary pre-metastatic niche formation. Breast cancer metastasis could occur through the accumulation of MDSCs within the lungs. These results provide new and promising avenues to develop practical therapeutic approaches for breast cancer treatment, especially TNBC.

### ER stress, a key regulator of LOX-1+ PMN-MDSCs derived from nasopharyngeal carcinoma survivors with chronic hepatitis B virus

Lectin-type oxidized LDL receptor-1 (LOX-1) is a specific marker for human PMN-MDSCs [7] that can separate and identify PMN-MDSC cells. CD15 is also a marker for neutrophils as such LOX-1+ and CD15+ cells in human blood are PMN-MDSC. In contrast, CD15+ but LOX-1− cells are normal neutrophils (PMNs) [29, 57, 84]. Levels of PMN-MDSC (LOX-1+) cells increased in the peripheral blood of nasopharyngeal carcinoma (NPC) survivors with chronic hepatitis B virus (CHB) infection. These cells may be immunosuppressive by inhibition of T cell proliferation and activation. ER stress-related gene: sXBP1, SEC61A, ATF4, ATF6, ATF3, and CHOP are significantly up-regulated in PMN-MDSCs (LOX-1+) compared with PMNs (LOX-1−) from the same NPC survivors with CHB. These observations suggest that ER stress may affect the survival of LOX-1+ PMN-MDSCs and disease progression. LOX-1+ PMN-MDSCs from NPC survivors with CHB had higher NOX2 mRNA levels, a critical ROS-related gene, suggesting that ROS mediates the immune suppressive effect of LOX-1+ PMN-MDSCs. These results suggest that PMN-MDSCs play an immunosuppressive role in the host immune response to CHB through ER stress/ROS effects [85].

### ER stress may be the key regulator of PMN-MDSCs in hepatocellular carcinoma patients

PMN-MDSCs (LOX-1+CD15+) is significantly up-regulated in the peripheral blood of hepatocellular carcinoma (HCC) patients compared to healthy controls. T cell activation is significantly suppressed by LOX-1+CD15+ PMN-MDSCs, inhibiting CD4+ and CD8+ T cell proliferation as well as IFN-γ production. This immune suppression is mediated by the cellular production of ROS and by the activation of arginase I. Moreover, LOX-1 expression and suppressive function are mediated by ER stress that increases the expression of XBP1, ATF3, and CHOP [86]. These results suggest ER stress may be an essential regulator of PMN-MDSC in HCC. In addition, PMN-MDSCs of cancer patients exhibit signs of an ER stress response [29, 87], with some myeloid cells in peripheral blood exhibiting ER stress. These peripheral blood cells were distant from the tumor site, which suggests tumor-induced ER stress in myeloid cells in a remote manner. However, neither serum nor the TCM from HCC patients induced healthy donor CD15 + cells to differentiate into PMN-MDSC, nor was ER stress-induced. The underlying mechanism for this phenomenon warrants further investigation [57].

### ER stress may mediate prostate cancer tumorigenesis by regulation of MDSC immune suppression

Prostate cancer is the most common urological malignancy in men, with three-quarters of cases in patients over 65. Compared with the United States, prostate cancer incidence and mortality are relatively low in China, although incidence and mortality have increased in recent years [88, 89]. The use of anti-CTLA-4 as an immune checkpoint blockade for prostate cancer treatment has not been clinically successful [90–92], which may be due to TME immunosuppression [93]. Myeloid-derived cells are essential components of the TME and may contribute to treatment failure in prostate cancer patients. Clinical studies have demonstrated increased numbers of infiltrating macrophages in primary prostate tumors, which may be associated with failure of androgen ablation [11]. The proportion of M-MDSCs in the peripheral blood of prostate cancer patients is significantly increased compared to age-matched controls [94, 95]. Mechanistically, T cell-suppressed proliferation, and high IL-10 levels have been confirmed in vitro [96]. Therefore, targeting MDSCs or regulating their recruitment has the potential for immunotherapeutic treatment of prostate cancer patients [97].

Recently, ER stress has been shown to be transmitted from tumor cells to myeloid cells. When cultured in the conditioned medium of ER-stressed tumor cells, macrophages also demonstrate an ER stress response with Hspa5 and XBP1 up-regulated. The proliferation of prostate cancer cell lines can be regulated by XBP1s [98], but how XBP1s regulate MDSCs is unknown and requires future investigation. ER stress-sensitive factor, XBP1, can induce the expression of Arg1 and Nos2, which are essential regulators of the immunosuppressive function of MDSCs [99]. ER stress may play an important role in prostate cancer, mediating tumorigenesis and tumor development by regulating the immunosuppressive phenotype of prostate cancer MDSCs [100, 101].

## ER stress and MDSCs as therapeutic targets in cancer and inflammatory disease

**Fig. 2:**
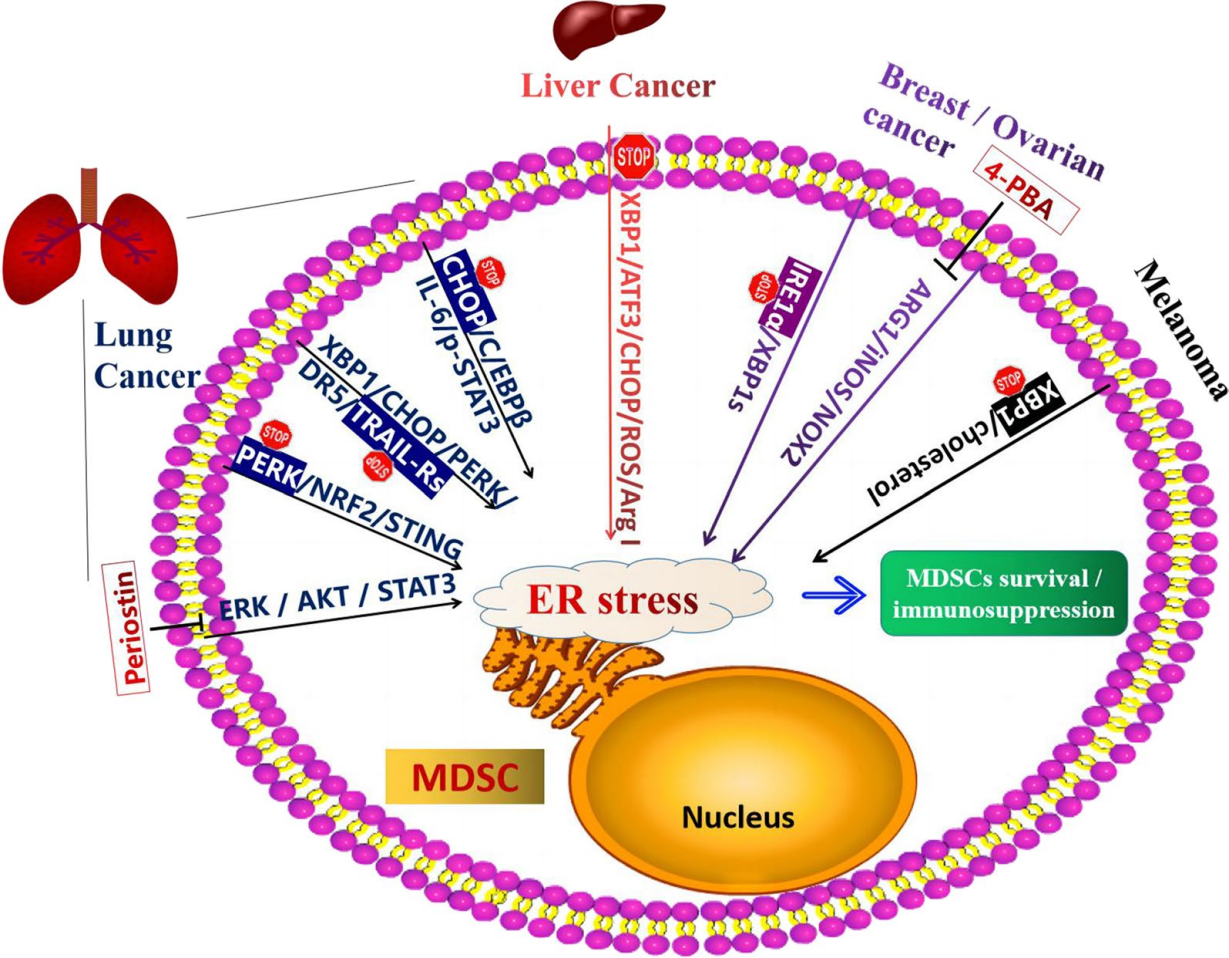
ER stress sensors or signals triggering MDSC activation and immunosuppression may serve as targets in immunotherapy in cancers. In lung carcinoma or metastasis cancer, CHOP, PERK, and TRAIL-Rs could be targeted by inhibitors that reduce MDSCs by inhibiting ER stress. sXBP1/ATF3/CHOP/ROS/Arg I signaling activated LOX-1+CD15+ PMN-MDSCs in Hepatocellular carcinoma. sXBP1 is one of the most important sensors in ER stress. Targeting sXBP1 signaling could reduce the immunosuppression of MDSCs in HCC and Melanoma. IRE1α, one of ER stress sensors, could also be an inhibitory target in Ovarian carcinoma. 4-Phenylbutyric acid (4-PBA), a chemical chaperone widely used as an ER stress reducer, attenuated Tg-induced MDSC expansion and tumor growth in melanoma

MDSCs play an essential role in tumor immunosuppression. More and more studies have shown that MDSCs are closely related to the effect of tumor immunotherapy. Therefore, it is of great significance to change tumor immunosuppression by inhibiting the function of MDSCs. Tumor-derived ER stress in MDSCs mediates the immunosuppressive activity. Therefore, researchers predicted ER stress-related proteins in MDSCs could be potential therapeutic targets in infectious diseases and cancers. ERK, AKT, and STAT3 decreased in Periostin (POSTN) -deficient MDSCs. The the pro-metastatic role of POSTN is limited to ER-negative breast cancer patients, which indicates that POSTN is a potential target for the prevention and treatment of breast tumor metastasis [91]. M10, a novel derivative of Myricetin, prevents ER stress-induced autophagy in inflamed colonic mucosal cells by targeting the NF-κB/IL-6/STAT3 pathway, which develops M10 as a promising regimen in the chemoprevention of colitis and colorectal cancer [66]. Insights from studies might substantiate PMN-MDSCs as a potential therapeutic target for lung carcinoma [97], hepatocellular carcinoma 6, and Chronic hepatitis B (CHB) with nasopharyngeal carcinoma (NPC) [85]. Further research were warranted to confirm ER stress-related proteins, including PERK, CHOP, IRE1α, and XBP1s, as potential therapeutic targets in cancers [102–104]. ER stress sensors or signals triggering MDSC activation could be investigated as therapeutic targets in cancers and infectious or inflammatory diseases as shown in Table 3; Fig. 2.

**Table 3:** ER stress sensors or signals triggering MDSC activation were investigated as therapeutic targets

	Diseases	ER stress sensors or signaling	Therapeutic targets or molecules	Types of activated MDSCs	MDSCs phenotype	Species	References
Cancer	Lung metastasis of breast cancer	ERK/AKT/STAT3	Periostin	MDSCs	a, b	Murine	[73]
	Lung carcinoma/lymphoma	IRE1ɑ/ATF6	Blockade of the ER stress response	PMN-MDSCs	a, b	Murine	[87]
	Lung carcinoma/ovarian carcinoma	PERK/NRF2/STING	PERK	MDSCs	a, b, c, d	Human/Murine	[91]
	Non small cell lung cancer/TB	XBP1/CHOP/PERK/DR5/TRAIL-Rs	TRAIL-Rs	MDSCs	a, b, d	Human/Murine	[12]
	Mice injected with 3LL lung carcinoma	CHOP/C/EBPβ, IL-6/p-STAT3	Chop	MDSC	CD11b^+^ Gr1^+^	Murine	[8]
	Triple-negative breast cancer	IRE1α/XBP1s	IRE1α kinase inhibitor	Cancer-associated fibroblasts	FAP	Murine	[72]
	HER2/CT26-bearing mice	ARG1/iNOS/NOX2	4-PBA	Ly6G + CD11b+ myeloid cells	Ly6G^+^CD11b^+^	Murine	[99]
	Ovarian carcinoma	IRE-1α/XBP-1	Targeting the ER stress response	Tumor-infiltrating DCs	Human (CD45^+^CD3^−^ CD20^−^CD11c^+^DEC205^+^) Murine (CD45^+^CD11c^+^CD11b^+^MHC-II^+^CD8a^low^)	Human/Murine	[3]
	Hepatocellular carcinoma	sXBP1/ATF3/CHOP/ROS/Arg I	LOX-1 + CD15 + PMN-MDSC	LOX-1 + CD15+ PMN-MDSC	LOX-1^+^CD15^+^PMN-MDSCs	Human	[86]
	T lymphocytes-based cancer	Ceramide/Cathepsin B/Cathepsin D/autophagy/ER stress/MDSC apoptosis- and necroptosis	LCL521	MDSCs	CD11b^+^ Gr1^+^	Murine	[103]
	prostate cancer	XBP1/Arg1/Nos2	XBP1	MDSC	c, d	Human	[89]
	Colitis and colorectal carcinogenesis	GM-CSF/M-CSF/IL-6/TNF-α/NF-κB/IL-6/STAT3	Naringin	MDSCs/tumor associated macrophages	a,b CD11b^+^F4/80	Murine	[70]
	Melanoma	XBP1/cholesterol	XBP1	MDSCs	CD11b^+^ Gr1^+^	Murine	[104]
Infection and Inflammatory Disease	Nasopharyngeal carcinoma with Chronic hepatitis B	ER stress/ROS	NOX2	LOX-1+ PMN-MDSCs	LOX-1^+^CD15^+^	Human	[77]
	Inflammatory bowel disease (IBD)	NOD2/ATG16L1/NALP3/chemokine (C-C motif) receptor 6	ER stress	MDSCs	c, d	Human	[102]
	Colitis	NF-κB/IL-6/STAT3	Myricetin 10	MDSCs	a,b	Murine	[66]
	TB and leprosy	IL-1α/IL-6/IFN-I/ATF6/IRE1/XBP1/PERK/ATF4	UPR elements of ER stress	MDSCs	c, d	Human	[92]
	leprosy	OLR1/XBP1/ATF4/ATF3/ERN1/IL-10	Recombinant IFN-γ	MDSC	HLA-DR^-^CD33^+^CD15^+^	Human	[54]

a: Murine-M-MDSCs (CD11b^+^Ly6G^−^Ly6C^hi^); b: Murine-PMN-MDSCs (CD11b^+^Ly6G^+^Ly6C^lo^); c: Human-M-MDSCs (CD11b^+^HLA-DR^−^CD14^+^CD15^−^); d): Human-PMN-MDSCs(CD11b^+^HLA-DR^−^CD14^−^CD15^+^)

## Future perspectives

The pathologic microenvironment associated with inflammation and tumors is characterized by hypoxia, nutrient deprivation, low pH, and free radicals that can trigger ER stress and the accumulation of MDSCs, resulting in immunosuppression. Reactive oxygen species and lipids are significantly elevated in MDSCs and are the main causes of the ER stress response. Inhibition of MDSC has been shown to be a potential and promising cancer therapy based on its complex role in promoting tumor genesis, development, and metastasis in the tumor microenvironment. Over the past few years, many preclinical studies have focused on exploring drugs, such as Sunitinib [105] and 5-phosphodiesterase inhibitors [106], to inhibit its immunosuppressive activity. New strategies that remodel tumor-associated myeloid cells into mature immune cells will greatly improved the efficacy of tumor-targeted therapies.
